# Effects of an online mindfulness-based cognitive therapy for caregivers of children with allergic rhinitis

**DOI:** 10.3389/fpsyg.2024.1372586

**Published:** 2024-08-14

**Authors:** Xixi Yan, Zhongwei Xiong, Huimin Sun, Jieli Li, Yadong Gao, Jinjin Zhang, Xiaomin Ding

**Affiliations:** ^1^Department of Allergy, Zhongnan Hospital of Wuhan University, Wuhan, Hubei, China; ^2^Department of Neurosurgery, Zhongnan Hospital of Wuhan University, Wuhan, Hubei, China; ^3^Department of Neuropsychology, Clinical Medical Research Center for Dementia and Cognitive Impairment of Hubei Province, Zhongnan Hospital of Wuhan University, Wuhan, Hubei, China; ^4^Department of Allergy, The First Affiliated Hospital, Zhejiang University School of Medicine, Hangzhou, Zhejiang, China

**Keywords:** mindfulness-based cognitive therapy, allergic rhinitis, caregiver, care burden, anxiety, depression

## Abstract

The incidence of allergic rhinitis in children is high across the world, as well as in China. Allergic rhinitis in children has serious impact on physical and mental health of the children. At the same time, the caregivers of allergic rhinitis children have heavy burden of care, and their mental problems are severe. It is necessary to implement timely psychological intervention for the caregivers of allergic rhinitis children. Mindfulness-Based Cognitive Therapy (MBCT) is a safe and effective psychological therapy, and the effect of online intervention can be comparable to the traditional face-to-face intervention program. This study focused on the mental health status in the main caregivers of children with allergic rhinitis, and conducted a modified online MBCT intervention on the caregivers, in order to improve their adverse mental state, meanwhile, improve the quality of care they provide. The results show that online MBCT intervention is applicable to the main caregivers of children with allergic rhinitis, and can effectively reduce caregiver burden, relieve anxiety and depression, and improve the level of mindfulness in the caregivers.

## 1 Introduction

Allergic rhinitis in children is a common chronic disease in childhood and adolescence in China, and it affects about 14% of children worldwide (Aït-Khaled et al., 2009; Li et al., 2011). Allergic rhinitis in children may cause many complications, including allergic asthma, allergic conjunctivitis and atopic dermatitis (Hellings and Fokkens, 2006; Siddiqui et al., 2022), which may seriously affect children’s sleep quality, learning and even growth and development. Since the main caregivers of the children with chronic disease take almost full responsibility for the care, in addition to daily care, caregivers also need to complete special care related to disease, including continuous supervision, treatment management and medication of the children (Drotar, 1981). These heavy care burden increase their stress, and the stress will have psychological, social, and even physical impact on the caregivers of the children with chronic disease (Zahr et al., 1994; Pinquart and Sorensen, 2003). Previous studies have well illustrated the anxiety and depression level of children with chronic diseases are significantly increased (van Oers et al., 2014). Thus, the mental health status of main caregivers of children with allergic rhinitis requires full attention.

There are worldwide studies targeting caregivers of children with chronic illness, which include skill trainings, environment changes, psychological interventions, physical excercises and group trainings (Sheng et al., 2019; Olij et al., 2021). Our study focus on the psychological interventions. The common psychological interventions which can elevate mental well-being include acceptance and commitment therapy (ACT), compassion interventions, cognitive therapy, expressive writing, mindfulness-based interventions (MBIs), positive psychological interventions (PPIs) and etc. In these interventions mentioned above, MBIs and PPIs can improve mental health of well-being across populations, including the general population and clinical population (van Agteren et al., 2021).

Mindfulness-based interventions (MBIs) have their roots in Buddhist tradition tracing back to 2,500 years ago. Systematic mindfulness-based therapy began in1979, Dr. Kabat-Zinn from the Massachusetts Medical School designed the Mindfulness-Based Stress Reduction (MBSR) therapy (Kabat-Zinn, 1990). Thereafter, MBIs have been integrated into various therapy, including Mindfulness-Based Stress Reduction (MBSR), Mindfulness-Based Cognitive Therapy (MBCT; Segal et al., 2002), Dialectical Behavior Therapy (DBT; Linehan, 1993) and Acceptance and Commitment Therapy (ACT; Hayes et al., 1999). With the rapid development of information technology, numerous psycho-therapeutic interventions, including MBIs, are conducted through the Internet. Accessible, available in participants’ own environment, anonymous and less costly, online MBIs is booming in the past decades via various electronic devices. Meta analyses have indicated the impact of online MBIs is compatible to the traditional face-to-face format on mental health outcomes, including stress, depression, anxiety and mindfulness (Spijkerman et al., 2016; Sommers-Spijkerman et al., 2021).

Mindfulness-Based Cognitive Therapy (MBCT) is a group-based intervention constructed by Teasdale et al., initially for prevention of relapses from depression (Segal et al., 2013). The practices of mindfulness in the MBCT program helps participants disengage with the patterns of negative thinking by increasing their awareness of their internal thoughts, emotions and bodily sensations at the present moment, together with a non-judgmental and accepting state of mind (Segal et al., 2013). Since the efficacy of MBCT first confirmed in 2000 (Teasdale et al., 2000), there has been accelerating interest in applying mindfulness in medical treatment (Sipe and Eisendrath, 2012). Besides clinical application in psychiatric disorders, cancer, pain, diabetes and etc., MBCT program has been used as a treatment to improve the mental health and well-being in the general populations, as well as the elderly population (Chiesa and Serretti, 2011; Querstret et al., 2018; Hazlett-Stevens et al., 2019; Ni et al., 2020; Pei et al., 2021; Lin et al., 2022). A RCT research demonstrated that MBCT can effectively alleviate the stress, anxiety and depression of the family caregivers of people with dementia (Kor et al., 2021). The application of MBCT is in its infancy in China, current limited Chinese studies show that MBCT can relieve anxiety, depression and other adverse emotions in various populations, including chronic heart failure patients, pregnant women, as well as breast cancer patients and improve their coping methods (Zhao, 2019; Wang et al., 2020; Zhao et al., 2020).

Our research focus on the mental health of the main caregivers of children with allergic rhinitis, the caregivers were given an 8-week online Mindfulness-Based Cognitive Therapy (MBCT). The purpose of our study was to implement intervention programs in order to explore the impact of the online MBCT program on care burden, mental status and mindfulness level of the main caregivers of children with allergic rhinitis.

## 2 Materials and methods

### 2.1 Study design

**Figure 1:**
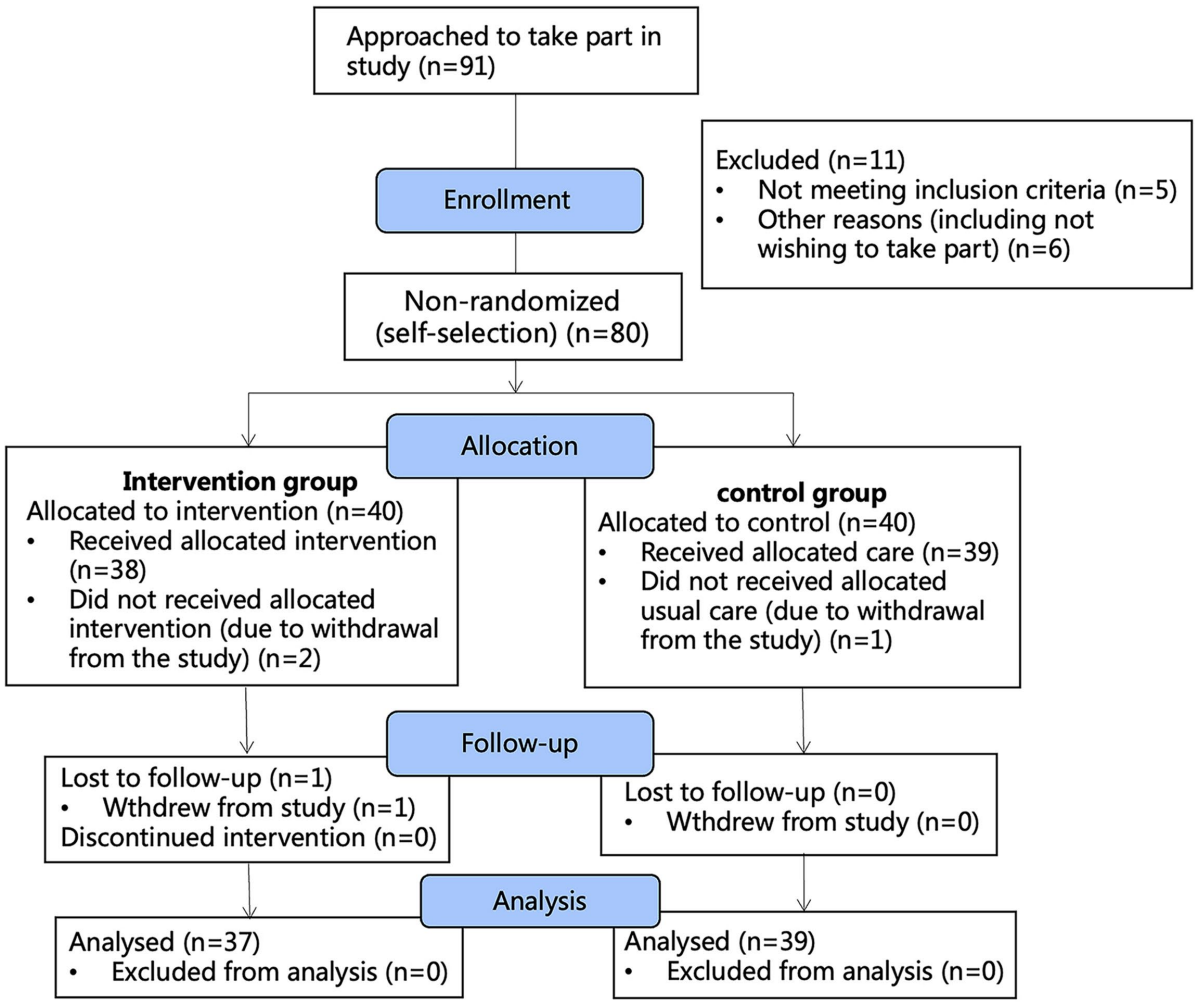
The study flow chart.

We used a quasi-experimental (non-randomized) concurrent control design with repeated measures and collected data pre- and post-intervention and at the 1-month follow up. Subjects was included in the study in strict accordance with the inclusion and exclusion criteria. Eligible main caregivers of children with allergic rhinitis was included in the study and divided into a control group or an intervention group according to their wishes, with a distribution ratio of 1:1. The control group received health education of allergic rhinitis as usual. The intervention group received a modified online MBCT intervention on the basis of usual health education that blinded recruiters and data collectors. This study was implemented online in the city of Wuhan, a TREND Checklist study flow chart is shown in Figure 1.

### 2.2 Setting and recruitment

Subjects was recruited from the allergy department of a tertiary hospital from September 2021 to March 2022 in Wuhan, the main city of central China, and offered science book of allergic diseases as gifts after the follow-up. Recruiter (XixiYan) presented this study to the main caregivers of children with allergic rhinitis who meet the study criteria, one by one, face- to-face and individually. If the caregivers were interested in participating in this study, they were asked to sign informed consent forms and assigned as they desired. The investigator explained to caregivers that this study would not adversely affect them and that participating caregivers could opt out of this study at any time. After the caregivers had joined in the WeChat group established for this study using their smartphone, the investigator discussed with the caregivers a suitable time for the online MBCT training each week to ensure every caregiver can participate.

### 2.3 Participants

The children were 5–14 years old, diagnosed of allergic rhinitis according to clinical practice guidelines (Brown, 2019), and under escalation phase of allergen specific immunotherapy (AIT) in the allergy department. The caregivers should accompany the children visit the allergy department once a week to receive injections during the escalation phase (Roche and Wise, 2014).

The screening criteria to guide the standard practice of MBSR, recommended by the Center for Mindfulness, University of Massachusetts Medical School USA (Santorelli, 2014) was used to formulate the inclusion and exclusion criteria of the participants in this study.

#### 2.3.1 Inclusion criteria for the main caregivers of children with allergic rhinitis

- Aged 18 years or above
- A fixed patient caregiver
- Live with the child, a parent or another immediate family member
- Providing most of the daily care and support for the child with allergic rhinitis (daily contact of at least 4 h or more)
- Good understanding and communication skills
- Using a smartphone
- Informed consent, willing to cooperate

#### 2.3.2 Exclusion criteria for the main caregivers of children with allergic rhinitis

- Having serious medical conditions
- Had learned or practiced mindfulness activities or meditation in the past 6 months
- Had been diagnosed with mental disorder or used psychotropic drugs
- Had self-reported suicidal thoughts in the past 6 months
- Taking care of other family member with chronic disease or disability at the same time

### 2.4 Sample size

**Figure 2:**
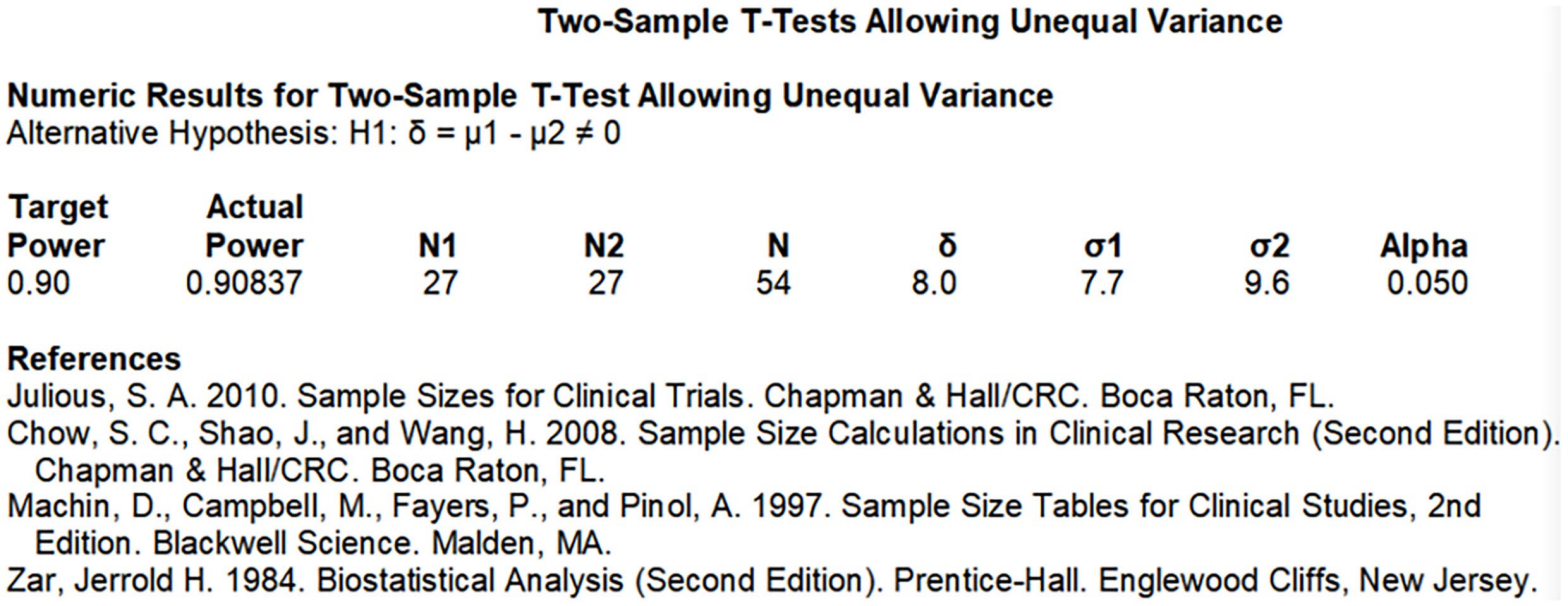
The sample size calculation process.

Sample size of this study was calculated by PASS 15. MBCT was constructed initially for prevention of relapses from depression, and based on a previous study results (Kor et al., 2021), depression status was the primary outcome measured by the Self-Rating Depression Scale (SDS). The mean SDS score in the intervention group decreased by 6.94 after intervention, and the standard deviation after intervention was 7.74. The mean SDS score in the control group increased by 1.03 after intervention period, and the standard deviation after was 9.61. Two-sided test level *α* = 0.05 was used, the degree of assurance is 1-β. The above parameters were put into PASS 15 software to estimate the sample size performing repeated measurement design ANOVA, 27 samples in each group (54 caregivers in total) were obtained for this study. In the actual study, the sample size was increased to avoid dropout, each group would need to include 40 caregivers (80 caregivers total) in the study. The definite calculation process is shown in Figure 2.

### 2.5 Intervention

#### 2.5.1 Control group

The control group received the usual health education on allergic rhinitis of children. The usual health education included the following: First, health education on allergic rhinitis provided by doctors and nurses during their visit to the department. Second, another WeChat group including all AIT patients had been established by the department before this study where doctors and nurses answered questions and shared disease-related knowledge with participants in this study, as well as other WeChat group members.

#### 2.5.2 Intervention group

The intervention group received a group-based, 8-session (2 h each) modified online MBCT program over 8 weeks. The program was implemented through a Chinese software: Tencent Conference, and contained various mindfulness practices (e.g., mindful meditation, body scanning and mindful eating) and peer sharing. The group size was 15–20 to insure sufficient interactions between the MBCT instructor and the caregivers. The instructor was a qualified MBCT teacher (Xixi Yan) who had received the MBCT training program developed by Oxford Mindfulness Centre, and obtained the certificate. The instructor was a postgraduate student in clinical psychology. In addition, the intervention was supervised by a senior psychotherapist (Huimin Sun). The MBCT was primarily established for people who suffered from recurrent depression, so we replaced the educational content related to depression with more appropriate knowledge and skills in allergic children care-giving. The main changes to the original MBCT protocol (Segal et al., 2013) included: eliminating the 8-h full-day retreat, incorporating teaching on mindfulness skills with care-giving task, including mindful communication with AR children, responding to negative moods resulting from care-giving mindfully, as well as identifying habitual emotional reactions to difficulties/ challenges in care-giving. The detailed arrangement of the intervention was shown in Table 1, and the intervention protocol was demonstrated in Supplementary Table S1 presentation.

**Table 1:** Detailed arrangement of the intervention.

**Item**	**Precise arrangement**
When	7:30 pm every Saturday night
Where	Tencent Conference online
Who	Participants met the inclusion criteria
Contact information	Mobile phone, WeChat, WeChat group
Trainer	The investigator
Training tools	Laptop, smartphone, Now mindfulness APP
Training method	Online teaching, home MBCT practice
Review	Upload the summary of training courses to the WeChat group
Home practice	Details in Supplementary Table S1
Training log	Self-practice record
Absence management	Self study the summary in the WeChat group, and practice following Now mindfulness APP

### 2.6 Instruments and measures

#### 2.6.1 Basic information

Basic information of the children with allergic rhinitis, including sociodemographic and clinical disease characteristics. Sociodemographic information included age and gender, clinical disease characteristics included disease course, allergic comorbidity and symptom scores. Symptom scores of allergic rhinitis was applied (Subspecialty Group of Rhinology, 2022). General information of the main caregivers included gender, age, education, employment, the caregiver’s relationship to the patient, the daily length of care and monthly family income.

#### 2.6.2 Outcome: caregiver burden

The Chinese version of Zarit Caregiver Burden Interview (ZBI) was used to assess the burden level of the research subjects (Wang et al., 2006). This scale is the main scale for evaluating the burden of caregivers in China at present stage. It has good reliability and validity in Chinese culture. The scale contains 22 items measured on a 5-point Likert scale, ranging from “0 = never” to “4 = always.” To score the scale, simply sum the scores of the 22 items, the higher the score is, the worse the level of caregiver burden. The cutoff value is 20 points: 21–40 points indicates mild burden; 41–60 moderate burden; greater than or equal to 61 severe burden. Cronbach’s alpha for this scale is 0.847 (Wang et al., 2006).

#### 2.6.3 Outcome: mood of anxiety

The Self-Rating Anxiety Scale (SAS) was used to assess the mood anxiety of the research subjects (Zung, 1971). This scale is suitable for adults with mood of anxiety in China, and convenient. It has wide applicability, good reliability and validity. The scale contains 20 items measured on a 1–4 point Likert scale, ranging from “1 = never” to “4 = always.” Add the scores for each of the 20 items to get the total rough score X, then multiply the rough score by 1.25 and take the integral part to get the standard score Y. The Chinese cutoff value is 50 points: 50–59 points indicates mild anxiety; 60–69 moderate anxiety; greater than or equal to 70 severe anxiety. Cronbach’s alpha for this scale is 0.777 (Wang, 1984).

#### 2.6.4 Outcome: mood of depression

The Self-Rating Depression Scale (SDS) was used to assess the mood depression of the research subjects (Zung, 1965). This scale is suitable for subjects with mood of depression in China, and convenient. It has wide applicability, good reliability and validity. The scale contains 2 dimensions and 20 items measured on a 1–4 point Likert scale, ranging from “1 = never” to “4 = always.” 10 items of the scale are worded positively, the remaining 10 items (2, 5, 6, 11, 12, 14, 16, 17, 18, 20) are worded negatively and reverse scoring. Add the scores for each of the 20 items to get the total rough score X, then multiply the rough score by 1.25 and take the integral part to get the standard score Y. The Chinese cutoff value is 53 points: 53–62 indicates mild depression; 63–72 moderate depression; greater than or equal to 73 severe depression. Cronbach’s alpha for this scale is 0.782 (Liu et al., 1999).

#### 2.6.5 Outcome: level of mindfulness

The Chinese version of Mindful Attention Awareness Scale (MAAS) was used to assess the level of mindfulness of the research subjects (Brown and Ryan, 2003; Siyi et al., 2012). This scale is suitable to measure subjects’ current level of awareness in China. It is convenient, and has good reliability and validity. The scale contains a single dimension and 15 items measured on a 1–6 point Likert scale, ranging from “6 = never” to “1 = always.” A lower score indicating a less sensitive awareness. To score the scale, simply sum the scores of the 15 items. Cronbach’s alpha for this scale is 0.89 (Chen et al., 2012).

### 2.7 Data collection

First, after obtaining informed consent from the subjects, the investigator took a few minutes to review possible challenges and respond to them based on experience with past mindfulness classes to reduce dropout rate. To maintain anonymity, each caregiver was given an identification number for their information and questionnaire. Then, baseline data was collected and be shown in Table 2. Measurement in two parallel groups were carried out before and after intervention, and at the 1 month follow-up by two trained investigators (Xiaomin Ding and Jieli Li) under the supervision of a psychologist (Huimin Sun). The clinical disease characteristics of the children with allergic rhinitis were extracted from their medical records, and supervised by a senior allergist (Yadong Gao). And a specified WeChat group including the intervention group of caregivers and the investigators was established. Baseline data included sociodemographic, clinical disease characteristics and psycho- social variables of the participants. Psycho-social variables included caregiver burden, level of anxiety, depression, and mindfulness. Investigators measured symptom scores of allergic rhinitis children and psycho-social variables of the main caregiver at three time points: baseline (T0), post- intervention (T1), and at 1-month follow-up (T2) (Table 3). Participants were assured that all data and intervention processes would be confidential. Only the main researchers had access to the final trial dataset. The data of participants who drop out in the midway was excluded.

**Table 2:** Baseline characteristics of participants.

Baseline characteristics	Total level (*n* = 80)	Intervention group (*n* = 40)	Control group (*n* = 40)	χ^2^/Test value	*p-*value
AR children					
Age	7.9 ± 2.4	8.1 ± 2.5	7.7 ± 2.3	−0.832	0.408
Gender (%)					
Male	38(47.5)	17(42.5)	21(52.5)	0.802	0.370
Female	42(52.5)	23(57.5)	19(47.5)		
Disease course(month)	6.5 ± 4.1	6.8 ± 3.6	6.2 ± 4.5	−0.572	0.569
Comorbidity	1.5 ± 0.6	1.5 ± 0.6	1.5 ± 0.7	0.350	0.728
Symptom score	10.5 ± 4.5	10.8 ± 3.9	10.2 ± 5.1	−0.640	0.524
Caregiver					
Age	39.2 ± 11.7	38.3 ± 10.7	40.0 ± 12.7	0.657	0.513
Gender (%)				0.487	0.485
Male	29(36.3)	13(32.5)	16(40.0)		
Female	51(63.7)	27(67.5)	24(60.0)		
Relationship (%)				0.442	0.516
Parents	69(86.2)	36(90.0)	33(82.5)		
Non-parents	11(13.8)	4(10.0)	7(17.5)		
Education level (%)				0.346	0.556
Below bachelor degree	14(17.5)	6(15.0)	8(20.0)		
Bachelor degree or above	66(82.5)	34(85.0)	32(80.0)		
Employment status (%)				0.845	0.655
Employed	59(73.7)	28(70.0)	31(77.5)		
Unemployed	13(16.3)	8(20.0)	5(12.5)		
Retired	8(10.0)	4(10.0)	4(10.0)		
Duration of care (h)/day(%)				0.134	0.935
<8	46(57.5)	23(57.5)	23(57.5)		
8 ~ 14	11(13.8)	5(12.5)	6(15.0)		
≥14	23(28.7)	12(30.0)	11(27.5)		
Monthly income Yuan (%)				0.346	0.556
<6,000	3(3.8)	1(2.5)	2(5.0)		
≥6,000	77(96.2)	39(97.5)	38(95.0)		
Psychosocial variables					
ZBI	29.6 ± 7.4	30.0 ± 7.4	29.2 ± 7.5	−0.496	0.621
SDS	53.5 ± 6.8	54.1 ± 6.4	52.9 ± 7.3	−0.799	0.427
SAS	36.3 ± 7.2	37.4 ± 6.5	35.2 ± 77	−1.365	0.176
MAAS	52.2 ± 6.9	51.4 ± 7.1	52.9 ± 6.7	0.409	0.327

AR, allergic rhinitis; ZBI, Zarit Caregiver Burden Interview; SDS, Self-Rating Depression Scale; SAS, Self-Rating Anxiety Scale; MAAS, Mindful Attention Awareness Scale.

**Table 3:** Pairwise comparison of outcomes between time points.

Groups	Outcomes	Baseline ①	T1 ②	T2 ③	*F-*value	*P-*value	Pairwise Comparison (*p-*value)
Control	ZBI	29.2 ± 7.6	26.8 ± 6.8	28.9 ± 6.4	1.304	0.275	*p*^①②^ = 0.143 *p*^①③^ = 0.870 *p*^②③^ = 0.193
	SDS	52.8 ± 7.3	53.5 ± 7.2	54.9 ± 8.1	0.785	0.458	*p*^①②^ = 0.686 *p*^①③^ = 0.221 *p*^②③^ = 0.411
	SAS	35.4 ± 7.8	36.8 ± 6.7	36.6 ± 6.0	0.505	0.605	*p*^①②^ = 0.349 *p*^①③^ = 0.439 *p*^②③^ = 0.869
	MAAS	53.2 ± 6.7	54.5 ± 8.8	54.9 ± 6.0	0.629	0.535	*p*^①②^ = 0.401 *p*^①③^ = 0.291 *p*^②③^ = 0.827
Intervention	ZBI	29.9 ± 7.7	24.5 ± 9.0	21.5 ± 4.4	12.868	0.000*	*p*^①②^ = 0.002* *p*^①③^ = 0.000* *p*^②③^ = 0.079
	SDS	54.2 ± 6.5	49.8 ± 8.1	45.4 ± 8.7	11.445	0.000*	*p*^①②^ = 0.017* *p*^①③^ = 0.000* *p*^②③^ = 0.020*
	SAS	37.2 ± 5.9	34.2 ± 8.7	33.0 ± 7.9	2.902	0.059	*p*^①②^ = 0.095 *p*^①③^ = 0.021* *p*^②③^ = 0.519
	MAAS	51.4 ± 7.3	56.6 ± 10.2	60.8 ± 13.1	7.504	0.001*	*p*^①②^ = 0.034* *p*^①③^ = 0.000* *p*^②③^ = 0.089

*p < 0.05.

### 2.8 Statistical analysis

Two investigators (Zhongwei Xiong and Jinjin Zhang) remotely reviewed and analyzed data. Analysis was performed using SPSS for Windows (version 26.0). Similarities in the baseline outcome variables between the two study groups were compared using an independent sample *t* (two-tailed) or Chi-square test. The *t-*test was used for comparison if the baseline data of the two groups are continuous variables, and the chi-square test was used for comparison if they were categorical variables. The effect of the intervention program was assessed by comparing changes in psycho- social variables in the intervention and control groups. Repeated measures ANOVA was used to assess the effect of the intervention at different time points between groups. *Post-hoc* pairwise comparisons was also performed to examine which pairs of time points (e.g., T0 – T1, T0 –T2, and T1 – T2) showed a significant difference in outcomes. The *p*-value of <0.05 was considered statistically significant.

## 3 Results

### 3.1 Baseline characteristics of the participants

Ninety-one caregivers of the children with allergic rhinitis were interested in participating in this study from September 2021 to March 2022. Of these, 86 met the study criteria and 80 of them agreed to participate in the study. These 80 participants were allocated into either the MBCT (*n* = 40) or control (*n* = 40) group by their self selection. The recruitment procedure was summarized in the TREND Checklist flow chart (Figure 1). Two batches of identical modified MBCT sessions were conducted over the study period, respectively from April to June and from June to August in 2022. And each session was consisted of 15–20 participants in each group.

The baseline characteristics of the children with allergic rhinitis between the two groups showed no significant difference. Meanwhile, there were no significant differences in baseline characteristics of the caregivers, too. Specifically, characteristics of the children with allergic rhinitis in the study included age, gender, disease course, comorbidity and symptom scores. And characteristics of the caregivers included age, gender, relationship, educational level, employment status, duration of daily care, monthly income, as well as psycho-social variables. The mean age of the children with allergic rhinitis was 7.9 (S.D. = 2.4) years, and the majority of the children were female (52.5%) (*n* = 42). The mean disease course of the children was 6.5 (S.D. = 4.1) years, the mean number of comorbidity was 1.5 (S.D. = 0.6), and the mean symptom scores of the children was 10.5 (S.D. = 4.5). Correspondingly, the majority of the caregivers were female (63.7%) (*n* = 51) and their mean age was 39.2 (S.D. = 11.7) years. The majority of the caregivers were parents of children with allergic rhinitis (86.2%) (*n* = 69). The results of baseline measurement indicated that the caregivers experienced mild level of caregiver burden (mean ZBI score = 39.6, S.D. = 7.4), mild level of depression (mean SDS score = 53.5, S.D. = 6.8), relatively low level of mindfulness (mean MAAS score = 52.2, S.D. = 6.9), and no mild level of anxiety yet (mean SAS score = 36.3, S.D. = 7.2).

### 3.2 Effects of modified online MBCT

**Figure 3:**
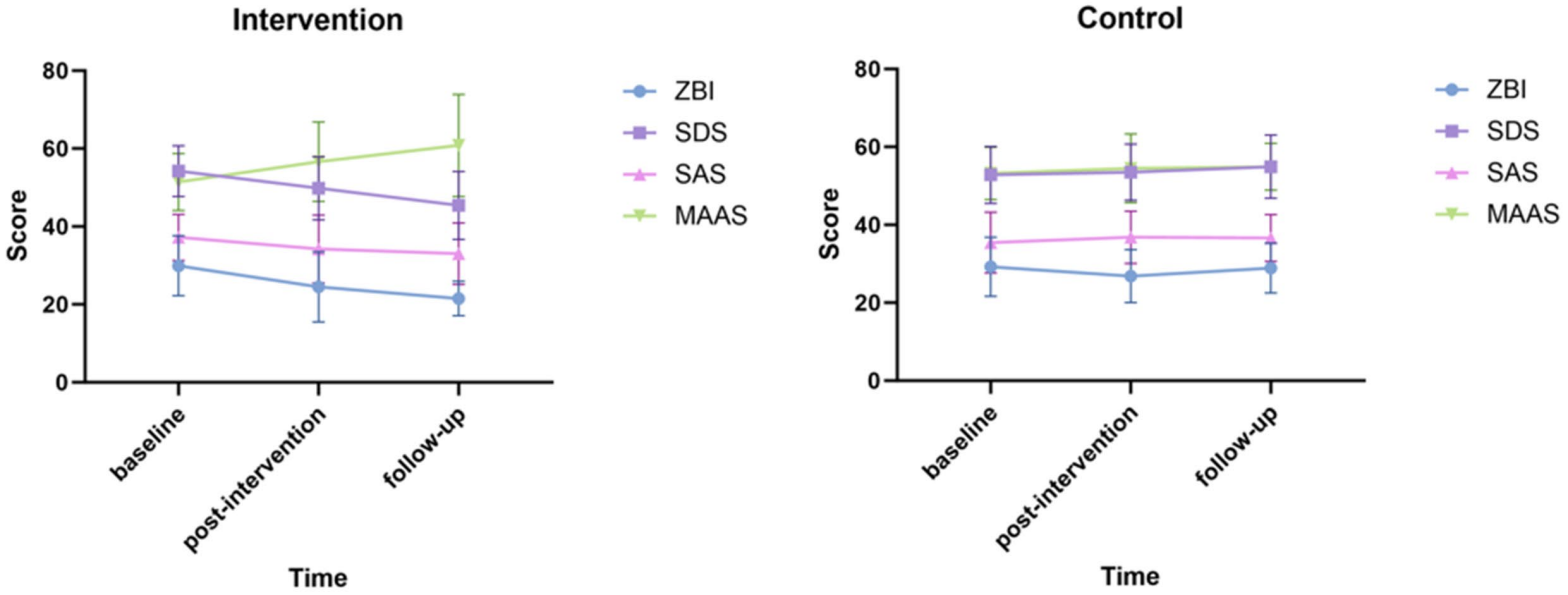
The outcomes of the two groups cross the three time points.

The outcomes of the modified online MBCT group and the control group across the three-time points (T0, T1, and T2) were summarized in Table 3, and visualized in Figure 3. On the whole, in the intervention group the caregivers showed significant improvement in the caregiver burden, mindfulness level, depression level, and anxiety level. Correspondingly, the control group had no statistically changed in these outcomes. At both T1 and T2, the intervention group had statistically improvement in caregiver burden (*p* < 0.05), depression level (*p* < 0.05) and mindfulness level (*p* < 0.05). In addition, significant greater improvements were demonstrated in caregiver burden (*p* < 0.001), depression level (*p* < 0.001) and mindfulness level (*p* < 0.001) in the intervention group from T2 to T0. Moreover, there was a statistically improvement in caregiver anxiety level from T2 to T0 (*p* < 0.05) in the intervention group. However, at both T1 and T2, there were no statistically improvement in caregiver anxiety level (*p* > 0.05) in the intervention group. In contrast, the control group had no statistically change in caregiver burden, depression, anxiety and mindfulness level from T1 to T0, T2 to T0, as well as T2 to T1 (*p* > 0.05). The post-hoc comparisons of all of the outcomes are summarized in Table 3, and presented in Figure 3.

### 3.3 Attrition rate

The overall attrition rate of the study was 5%. And the attrition rate of the modified online MBCT group and the control group was 7.5% and 2.5%, respectively. In the intervention group, 2 participants did not complete the intervention, one due to the lack of time, and another due to relocation. In the control group, only 1 participant withdrawn from the study due to the lack of time. The data of the attrited subjects mentioned above was eliminated from the analysis of the post-hoc comparisons.

## 4 Discussion

To date, this is the first quasi-experimental concurrent controlled trail to examine the effects of a modified online MBCT intervention on the caregivers of children with allergic rhinitis. Meanwhile, this is the first clinical practice in China to intervene patient caregivers with a MBCT program. We compared with a previous study of the modified MBCT program on dementia caregivers with attrition rate of 8.9% (Kor et al., 2021), the modified online MBCT protocol in this study resulted in a lower attrition rate of 5%. The caregivers of allergic rhinitis children demonstrated a significant improvement in the caregiver burden and mindfulness level, but the effect lasted no more than a month after the intervention. In addition, depression level of the caregivers improved significantly, and the effect could last until the 1-month follow-up.

Primarily, once diagnosed with allergic rhinitis, children need to endure long-term medication, frequent medical visits, physical discomfort, and complications. Meanwhile, the caregivers of the children have to withstand great caregiver burden, either from the physical, psychological, emotional, or from other aspects. The occurrence of children’s disease makes the caregivers be under psychological stress, and this stress response and coping style will inevitably affect the level of care they provided. We are able to anticipate that a program which can reduce the caregiver burden of the caregivers in children with allergic rhinitis, will have positive effect on promoting the physical and mental health of the children with allergic rhinitis, as well as their caregivers. In our study, the traditional MBCT therapy (Segal et al., 2013) was modified in accordance with the specific need of the caregivers, and performed in a relatively safe environment the caregivers had chosen. The caregivers were encouraged to discuss their care-giving experiences that increase caregiver burden, and their coping mechanisms were enhanced through mindfulness practice. The result of our study was consistent with the previous study conducted by Wood et al. (2015), the modified MBCT is appeared to be an effective intervention for caregiver burden reduction of chronic disease patients’ caregivers.

Next, it is clear that MBCT is an effective treatment for recurrent depression, anxiety and other problems which can cause stress. As the number of depression recurrence increases, there is a growing “intrinsic susceptibility,” an overreaction to adverse emotion, which manifests itself in two ways: experiential avoidance, an effort to avoid unwanted experiences; rumination, to solve the problem of mind in a exhausted way. Therein, rumination involves a repetitive and passive focus on the causes, consequences and symptoms of negative care-giving experiences, is an important mediator of depression and anxiety in patient caregivers (Nolen-Hoeksema, 2000; Slavish, 2015). The modified MBCT program aims to eliminate the mediator, helps the caregivers disengage from their ruminative thoughts through mindfulness practice, and works around coping “intrinsic susceptibility.” It focuses on the participants building new relationships with individual negative care-giving experiences, rather than changing the experiences themselves, thus, the caregivers will respond more skillfully to these negative care-giving experiences and mental process. Combining the observation of our study and a previous study (Kor et al., 2021), it can deduce that the modified MBCT therapy can effectively reduce anxiety and depression in the caregivers of patients with chronic disease.

Moreover, our study monitored mindfulness level of the participants for almost 3 months, before and after the intervention, and at the 1-month follow-up. The results showed that the modified MBCT therapy can significantly improve mindfulness level in the caregivers of patients with chronic disease, which is consistent with the results of a previous study (Kor et al., 2021). Furthermore, to explore the factors which may influence the effects of MBCT therapy, future research can make a more detailed analysis of the differences between the participants who benefit more and the participants with no apparent effects.

Finally, in 2019, a study from Birtwell et al. (2019) showed that improvement in a participant’s mindfulness level resulted not only from formal mindfulness practice, but also from informal mindfulness practice. The informal mindfulness practice refer to mindfulness practice which has been incorporated into the daily activities of the participants, such as mindful walking and mindful eating. Formal practice and informal practice of mindfulness reinforce and complement each other, and foster the same kind of mindfulness. Formal practice of mindfulness is like sharpening a knife. Informal practice of mindfulness in daily activities is like using a life. Along with the learning process, we need to understand how to diffuse the gentle awareness fostered by formal practice of mindfulness into our daily lives, in order to be better aware of and response to adverse care-giving experiences. As Dr. Kabat-Zinn has said, “the real Session 8 is the rest of our lives” (Segal et al., 2013), we should not regard the last session as the ending, but rather as the starting of a broader and ongoing journey of mindfulness. In our study, we did not document the informal practice of mindfulness of the caregivers and how they incorporated mindfulness practice into their care-giving routines. Future research can record and analyze the influence of informal practice mindfulness practice on the MBCT intervention effect, expand the scope of MBCT intervention, thereby, help the caregivers maximize the use of mindfulness in the process of caring for patients with chronic diseases and improve their care-giving experiences.

## 5 Conclusion

In summary, the modified online MBCT program helps to equip the main caregivers of children with allergic rhinitis with the skills of mindfulness to improve their care-giving experiences. The 8 week intervention appears to be effective at reducing their care-giving burden, relieving their anxiety and depression level, and improving their mindfulness level. Comparing to the traditional MBCT therapy, the modified online MBCT program is more convenient and less restricted to the site. In addition, it has strong operability, and good intervention effect, which is suitable for clinical practice. It is recommended that future research be conducted in different medical settings, recruit participants with a wider range of sociodemographic backgrounds, and investigate more psychological indicators to further verify the efficacy.

## Data availability statement

The raw data supporting the conclusions of this article will be made available by the authors, without undue reservation.

## Ethics statement

The studies involving humans were approved by Ethics Committee of Zhongnan Hospital of Wuhan University. The studies were conducted in accordance with the local legislation and institutional requirements. The participants provided their written informed consent to participate in this study.

## Author contributions

XY: Conceptualization, Formal analysis, Methodology, Project administration, Software, Validation, Writing – original draft, Writing – review & editing. ZX: Data curation, Formal analysis, Software, Visualization, Writing – review & editing. HS: Conceptualization, Investigation, Methodology, Supervision, Validation, Writing – review & editing. JL: Formal analysis, Resources, Validation, Writing – review & editing. YG: Conceptualization, Investigation, Methodology, Validation, Writing – review & editing. JZ: Data curation, Formal Analysis, Software, Visualization, Writing – review & editing. XD: Formal Analysis, Resources, Validation, Writing – review & editing.
