# Supplementary material for: Effects of an online mindfulness-based cognitive therapy for caregivers of children with allergic rhinitis
Source: Front Psychol. 2024 Aug 14;15:1372586. doi: 10.3389/fpsyg.2024.1372586 (PMC11349699; doi:10.3389/fpsyg.2024.1372586)
Supplement: Supplementary file 1 [file Table_1.DOC]

Table S1. Protocol of the modified mindfulness-based cognitive therapy program

| Protocol of the modified mindfulness-based cognitive therapy program | | | |
| --- | --- | --- | --- |
| Session | Main Theme | Contents | Home practice |
| 1 | Awareness and automatic pilot | - Self introduction of the instructor and caregivers and establishing the orientation of the class - The Raisin exercise (eating meditation) - 45-min body scan - Feedback and discussion of the exercise - Distribute audio files and Session 1 participant handouts (including the Home Practice Record form) via the WeChat group | - Emphasizing the importance of home practice - 45-min body scan for 6 out of 7 days - Mindfulness of a routine practice |
| 2 | Living in our heads | - Practice review and encourage persistence practice - Exercises on thoughts and feelings related to care-giving - 45-min body scan - Brief mindfulness of breathing | - 45-min body scan for 6 out of 7 days - 10 min of mindfulness of breath for 6 out of 7 days - Pleasant experience calendar (one example daily) - Mindfulness of a routine practice |
| 3 | Gathering the scattered mind | - Practice review - Exercises focusing on unpleasant experiences resulted from care-giving - 5-minute practicing seeing or hearing - 30-minute sitting meditation (awareness of breath and body, and of responses to painful sensations) - 3-min breathing space - Mindful stretching and breath meditation | - Stretching and breathing meditation on Days 1, 3, and 5 - 40 min of mindful movements on Days 2, 4, and 6 - Unpleasant experience calendar (a different experience for each day) - 3-min breathing space, 3 times daily |
| 4 | Recogni-  -zing aversion | - Practice review - 5-minute practicing seeing or hearing - Mindful communication with child with AR - 3-min breathing space - Mindful walking - 30-minute sitting meditation (awareness of breath, body, and sounds, then of how we relate to our experience through the reaction) | - Sitting meditation, 6 out of 7 days - 3-min breathing space-regular (3 times a day) - 3-min breathing space – responsive (whenever we notices unpleasant feelings) |
| 5 | Allowing/Letting be | - Practice review - 30-minutes sitting meditation - Exercises on thoughts and alternative viewpoints - 3-min breathing space (responsive) - Read Rumi’s poem”The Guest House” | - Select a guided meditation to practice at least 40 min per day - 3-min breathing space-regular (3 times a day) - 3-min breathing space – added instructions (whenever one notices unpleasant feelings) |
| 6 | Thoughts are not  facts | - Bravo for our persistence - Practice review - 30-minutes sitting meditation with breath, body, sounds - Activity and mood exercise - Identifying habitual emotional reactions to difficulties resulted from care-giving - 3-min breathing space (responsive) | - Practice with a selection of guided meditations for a minimum of 40 minutes a day. - 3-min breathing space-regular (3 times a day) - 3-min breathing space – responsive (whenever one notice unpleasant feelings) |
| 7 | How can I best take care of myself ? | - Practice review - 30-minutes sitting meditation- awareness of breath and body - Plan how best to schedule activities for when mood threatens to overwhelm - 3-minutes breathing space or mindful walking | - 3-min breathing space -regular (3 times a day) - 3-min breathing space –responsive (whenever one notices unpleasant feelings) - Select from all different forms of practice and apply them on a regular basis - Develop actions to be used in the face of low moods |
| 8 | Maintain-  -ing and extending new learning | - Express gratitude for accomplishing the class - Exercise on looking forward - Exercise on preparing for the future |  |
